# Automated methodology for optimal selection of minimum electrode subsets for accurate EEG source estimation based on Genetic Algorithm optimization

**DOI:** 10.1038/s41598-022-15252-0

**Published:** 2022-07-02

**Authors:** Andres Soler, Luis Alfredo Moctezuma, Eduardo Giraldo, Marta Molinas

**Affiliations:** 1 Department of Engineering Cybernetics, Norwegian University of Science and Technology, Trondheim, Norway; 2 Department of Electrical Engineering, Universidad Tecnológica de Pereira, Pereira, Colombia

**Keywords:** Neuroscience, Biomedical engineering

## Abstract

High-density Electroencephalography (HD-EEG) has proven to be the EEG montage that estimates the neural activity inside the brain with highest accuracy. Multiple studies have reported the effect of electrode number on source localization for specific sources and specific electrode configurations. The electrodes for these configurations are often manually selected to uniformly cover the entire head, going from 32 to 128 electrodes, but electrode configurations are not often selected according to their contribution to estimation accuracy. In this work, an optimization-based study is proposed to determine the minimum number of electrodes that can be used and to identify the optimal combinations of electrodes that can retain the localization accuracy of HD-EEG reconstructions. This optimization approach incorporates scalp landmark positions of widely used EEG montages. In this way, a systematic search for the minimum electrode subset is performed for single- and multiple-source localization problems. The Non-dominated Sorting Genetic Algorithm II (NSGA-II) combined with source reconstruction methods is used to formulate a multi-objective optimization problem that concurrently minimizes (1) the localization error for each source and (2) the number of required EEG electrodes. The method can be used for evaluating the source localization quality of low-density EEG systems (e.g. consumer-grade wearable EEG). We performed an evaluation over synthetic and real EEG datasets with known ground-truth. The experimental results show that optimal subsets with 6 electrodes can attain an equal or better accuracy than HD-EEG (with more than 200 channels) for a single source case. This happened when reconstructing a particular brain activity in more than 88% of the cases in synthetic signals and 63% in real signals, and in more than 88% and 73% of cases when considering optimal combinations with 8 channels. For a multiple-source case of three sources (only with synthetic signals), it was found that optimized combinations of 8, 12 and 16 electrodes attained an equal or better accuracy than HD-EEG with 231 electrodes in at least 58%, 76%, and 82% of cases respectively. Additionally, for such electrode numbers, lower mean errors and standard deviations than with 231 electrodes were obtained.

## Introduction

Since the emergence of the international 10–20 system of electrode placement in 1958^1^, electroencephalography (EEG) systems have gradually evolved towards high-density EEG (HD-EEG). This first began with the 10–10 system^2,3^, then with geodesic electrode distribution to increase the number up to 256 channels^4^, and ultimately with the 10–5 system^5^ which sets the maximum number of EEG electrode positions at 345 locations for scalp coverage. The 10–10 system was accepted as standard by the American Clinical Neurophysiology Society and the International Federation of Clinical Neurophysiology^6,7^, yet the 10–5 system was not accepted by either organization^8^. The 10–10 system and the 10–5 system were developed as extensions of the 10–20 system contemporaneously with the development of EEG source localization techniques which required a higher density of electrode settings to reduce localization errors.

Accurate EEG source localization depends on many factors, with the most important including the density and location of scalp electrodes. It is widely accepted that an increased density of EEG electrodes generally provides more precise localization^9–11^. Today, 128 EEG channel systems and even 256 EEG channel systems are common commercial choices^5,12^. Even though the use of HD-EEG systems has resulted in improved spatial resolution in source localization^13^, practical use has come at a cost^8,14^.

In an extensive investigation of the validity of the 10–20, 10–10 and 10–5 systems as relative head-surface-based positioning systems^14^ it has been argued that even though the 10–20 system has not been conceived to support localization of brain sources, its high-density extensions into the 10–10 and 10–5 systems have mainly provided increased electrode density that has proven to be effective in brain source localization^13^. The high-density extensions of the 10–20 system logically inherit its electrode positioning principle, which was not conceived to improve the accuracy of brain source localization algorithms. Although it has been sufficiently proven that these extensions are effective in increasing the accuracy of brain source localization, it remains to be seen to what extent these high-density systems require all the electrode positions for attaining that level of accuracy. Increasing the number of electrodes decreases the localization error, but this improvement plateaus at some point^15^. This was concluded by examining the relationship between localization error and the number of electrodes when examining partial epilepsy in pediatric patients^15^. The same is true, however, for every source/es being examined. In this regard, the questions we attempt to answer in this paper are: what is the minimum number of electrodes required for an accurate source localization of a particular brain activity? and, is it possible to identify optimal low-density subsets of electrodes that can retain the source reconstruction quality of HD-EEG?

Here we propose an automated method that uses information from electrode locations on multiple systems and electrode configuration (e.g. the systems inherent to the 10–5, 10–10, 10–20 and/or geodesics systems) to select the optimal number of electrodes and their locations to solve the problem of EEG source localization for single and multiple sources. The optimization is based on the non-dominated sorting genetic algorithm II (NSGA-II)^16^. The algorithm, combined with EEG inversion methods, searches for combinations of electrodes to solve the EEG inverse problem that minimizes the localization error while using the lowest number of channels possible. Considering *C* as the number of channels, exploring all the combinations of electrodes in order to find the optimal solution means solving the inverse problem $2^C-1$ times for a single source case, and $s(2^C)-1$ for *s* multiple sources. These numbers will grow exponentially when increasing the number of channels, significantly increasing the computational efforts. For example, in the case of 128 electrodes, it is required to solve the inverse problem 3.4d38 times to evaluate all possible electrode combinations. In contrast, the NSGA-II aims to reduce the computational cost on average to $O*(P^2)$, where *O* is the number of objectives, in this case $s+1$, and *P* the population size^16^. NSGA algorithms have been successfully applied in multiple fields for optimization and feature selection, such as face expression recognition^17^ and telecommunications^18^. This algorithm has also been applied to EEG channel selection for classification of motor imagery^19^. Moreover, the algorithm has been proven to be effective in identifying low-density EEG subsets that maximize classification accuracy while reducing the number of EEG channels required for epileptic seizure classification^20^ and subject identification^21^.

Multiple works have evaluated sparse arrays of electrodes for source reconstruction. In one study, seven channels were used for source reconstruction on real and synthetic EEG signals^22^, where localization errors between 12 to 16 mm were found by using Multiple Sparse Priors (MSP) as inverse solver. However, no information about the electrode selection criteria was mentioned. In another paper, pre-preprocessing with Multivariate Empirical Mode Decomposition (MEMD) was successfully combined with MSP to improve the source reconstruction with low-density electrode arrays of 32, 16 and 8 channels on real and synthetic EEG signals^23^. The subset of electrodes was selected based on spatial coverage of the brain, but it was not optimized according to the source activity. In another study, a methodology based on regions of interest was proposed^24^, where relevance analysis and electrode distance to the regions were the criteria for selecting the subset of electrodes when solving the EEG inverse problem. The low-density subsets were combined with MSP and standardized low-resolution tomography sLORETA, obtaining localization errors below 10 mm by using the 4, 8 and 16 most relevant electrodes from a high-density set of 60 channels. The error levels are remarkable and comparable to high density levels. However, the study was limited by its evaluation on only synthetic EEG signals.

In this work, we approach the question of electrode reduction in a step-by-step manner. First, we formulate a computer simulation-based source reconstruction problem for one single source and for multiple sources. In a second phase, we examine how effective this automatic methodology is in accurately detecting the stimulation site from intra-cerebral stereotactically implanted electrodes, based on the dataset recorded by Mikulan et al.^25^. For our study we combined the NSGA-II algorithm with three widely used source reconstruction algorithms, the weighted minimum norm estimation (wMNE), the sLORETA, and the MSP, with a particular emphasis on studying the effect of optimally reducing the number of electrodes, and investigating their locations for a specific source activity.

## Materials and methods

### Methodology for channel selection based on genetic algorithm multi-objective optimization

**Figure 1:**
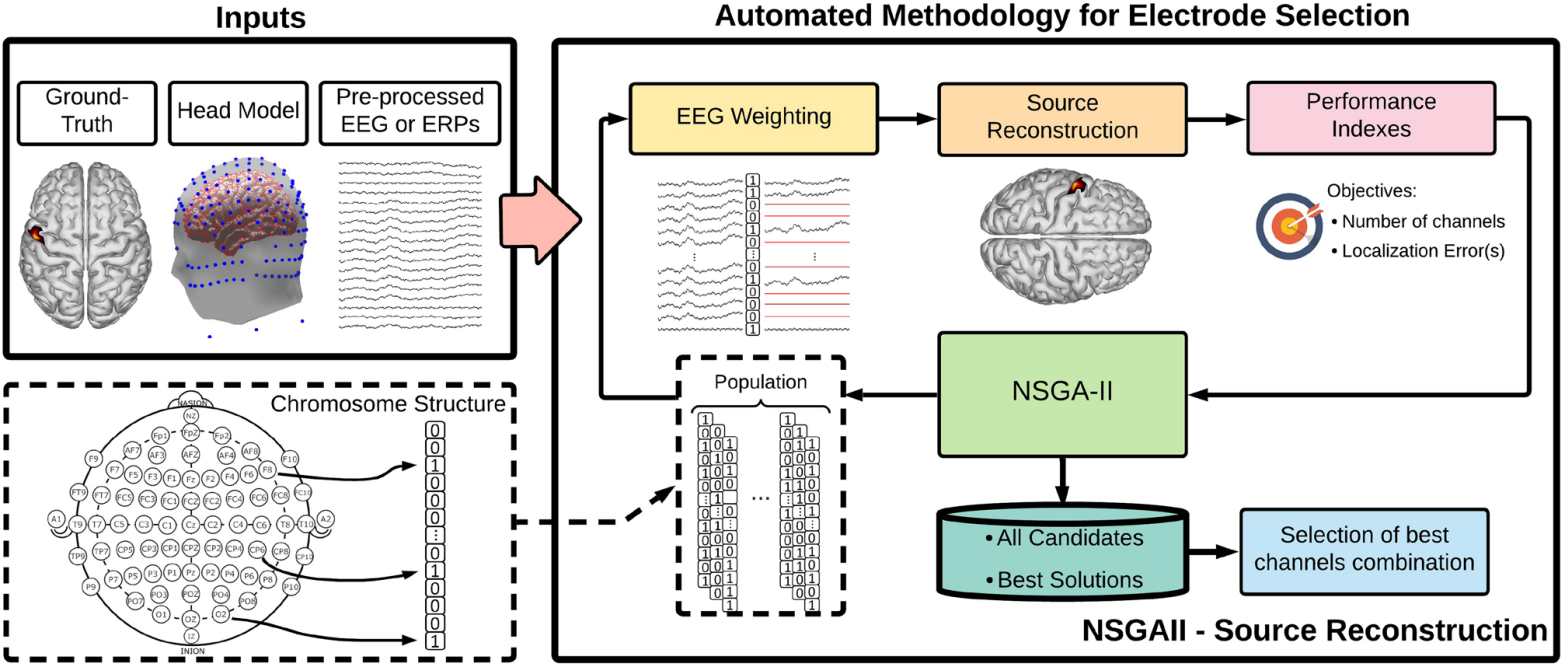
Flowchart of the proposed automated methodology for channel selection based on genetic algorithm multi-objective optimization.

EEG source reconstruction refers to the estimation of the properties of the source activity (source location, direction, and waveform) from the information registered by the electrodes on the scalp. To estimate this, the EEG inverse problem must be solved (the inverse problem is presented in more detail in “EEG forward and inverse problems” section). The proposed methodology combines a source reconstruction algorithm and the NSGA-II^16^, where the algorithm objective is to find electrode subsets with the minimum number of channels that retain the highest possible source localization accuracy (the accuracy of the reference HD-EEG). For the optimization process several inputs are required: The EEG or the event related potentials (ERPs) of the source(s) to analyze, the head model (this is required for source reconstruction, see “EEG forward and inverse problems” section) and the ground-truth location of the source activity to calculate the localization errors. The general structure of the automated methodology is presented in Fig. 1. The process can be summarized by a loop of four blocks: NSGA-II, Weighting, Source Reconstruction, and Performance indexes, where the outputs of NSGA-II are a set of best channel combinations and a set of all candidates.

An optimization problem consists of maximizing or minimizing a function by systematically choosing input values from a valid set and computing the value of the function, which can be limited to one or more constraints or can have no restrictions. In an optimization problem, the model is feasible if it satisfies all the restrictions, and it is optimal if it also produces the best value for the objective function. In the case of a Multi-objective optimization problem (MOOP), it has two or more objective functions, which are to be either minimized or maximized. As in a single-objective optimization problem, a MOOP may contain a set of constraints, which any feasible solution must satisfy^26^. In a multi-objective optimization problem, there is a set of solutions that is superior to the others in the search space when all the objectives are considered, but inferior to the other solutions for one or more objectives. Such solutions are known as Pareto-optimal solutions or *non-dominated solutions* and the rest as dominated solutions. The non-dominated sorting ranking selection method is used to emphasize good candidates and a niche method is used to maintain stable sub-populations of good points. NSGAs were created based on this concept^27^.

The central block of NSGA-II consists of several stages: Population initialization, Fitness calculation, Crossover, Mutation, Survivor selection, and Termination criteria to return the best solutions. The population consists of a set of chromosomes, which are possible solutions to the problem, and each chromosome can have as many genes as variables in the problem. In this case, each gene represents an EEG channel and the chromosome contains as many genes as the number of EEG channels in the registered EEG, represented by ${{\varvec{y}}}$. A chromosome structure representing the electrode combination is presented at the bottom-left of Fig. 1. The NSGA-II initializes with a population of *P* chromosomes with random binary values. Then, in the weighting block, the gene values are used to weight the EEG by a dot multiplication between the EEG and the chromosome to compute the weighted EEG ${{\varvec{y_{w}}}} = {{\varvec{y}}} \cdot chromosome$. As a result of this mathematical operation, the channels with a gene value of one keep their value, while information of the channels with a gene value of zero is discarded.

The algorithm is defined to minimize **the number of EEG channels** used to localize single or multiple sources, as a fixed objective. Minimizing **the localization error** of a number *s* of desired sources to localize is defined as a subsequent separate objective, one per each source. Therefore, the number of objectives *O* is defined as $O=s+1$. NSGA uses a non-dominated sorting ranking selection method to emphasize good candidates and a niche method to maintain stable sub-populations of good points (Pareto-front), where a non-dominated solution is a solution that is not dominated by any other solution^27^.

The weighted EEG ${{\varvec{y_{w}}}}$ is used in the source reconstruction block for calculating the source activity and estimating the location of each source. In each weighted EEG ${{\varvec{y_{w}}}}$ the non-selected channels are converted to a temporal data series with zero value. Therefore, the inverse solution is calculated using all the electrodes available in the head conduction model, but the information contained in the non-used electrodes is discarded during source estimation. The use of all electrodes is preferred to avoid increasing the ill-posedness of the inverse problem when using a subset of the volume conduction matrix and to avoid using computational resources for re-calculating the volume conduction for each candidate combination.

In the performance indexes block, the number of channels extracted from each chromosome and the localization errors per each source are computed by comparing the estimated position with the ground-truth. The performance indexes return to the NSGA-II block that applies the non-dominated sorting, and half of the population with better performance is used to create the next generation using crossover and mutation procedures. For this, NSGA-II uses Simulated Binary Crossover (SBX) and Polynomial mutation, with crossover probability as 0.9 and mutation probability 1/*n*, where n is the number of decision variables^27,28^. The actual generation finishes when a new population is created and the process is repeated with the next generation until the termination criterion is reached. We used a maximum number of generations as criterion to stop the algorithm.

Finally, from the all set of chromosomes, the best combination per each number of channels is extracted to create a pseudo-Pareto front. In the case of the multiple sources, the pseudo-Pareto front is generated from the mean of the localization errors of all the sources.

### EEG forward and inverse problems

The electrical activity recorded by the electrodes and its relation with the sources at cortical areas can be represented by the following equation, known as forward problem equation^29^:

1 $${{\varvec{y}}}={{\varvec{M}}}{{\varvec{x}}}+\varepsilon$$

The forward problem allows us to compute the potentials registered by the electrodes in the scalp that were produced by current sources in the brain cortical areas. In the forward problem equation ${{\varvec{y}}}\in \mathbb {R}^{d\times K}$ represents the signals recorded by *d* number of electrodes in *K* time samples; and ${{\varvec{x}}}\in \mathbb {R}^{n\times K}$ represents the time course activity of *n* sources at the cerebral cortex. The forward model ${{\varvec{M}}}\in \mathbb {R}^{d\times n}$ represents the relationship of the cortical source activity ${{\varvec{x}}}$ with the electrode measurements ${{\varvec{y}}}$. This matrix is also known as lead field matrix or volume conductor model. In the EEG case, ${{\varvec{M}}}$ represents how the electrical field propagates from the current sources in the brain to the scalp, where the voltages are registered by the electrodes. The variable $\varepsilon$ represents the noise in the measurements. It is assumed to follow a normal distribution with zero mean.

The inverse problem is the estimation of the current sources in the brain from the electrical potential registered by the electrodes. Computing the sources from the electrode information is an ill-posed problem due to the fact that the number of sources to estimate are much higher than the number of electrodes. An infinite number of combinations of sources could lead to the same recordings obtained by the electrodes. In addition, the problem is also ill-conditioned because the solutions are very sensitive to noise, and low perturbations on the channels can highly distort the source estimation^30^.

#### Source reconstruction algorithms

One of the factors that influences the accuracy of source localization is the inverse algorithm used. For this reason, and to consider this influence, we have selected three widely used algorithms to solve the inverse problem: wMNE^31,32^, sLORETA^13^, and MSP^33^.

wMNE is a variant of the original minimum norm estimation (MNE) based on Tikhonov regularization^31,34,35^. wMNE involves a weighting matrix to compensate for the distance of the deep sources to the electrodes^13,36^. sLORETA is a well-known method recognized by its zero localization error in the absence of noise^37,38^. This method is based on minimum norm, and introduces an improvement by standardizing the solution using the variance of the estimated activity^13^. MSP is based on a Bayesian approach, and utilizes a set of priors over the cortical sheet to optimize hyper-parameters related to the covariance of the source activity^33,39^. This method offers a focalized (less blurry) estimation of the source activity.

#### Source reconstruction error measurements

The localization error is defined as the euclidean distance between the position of the ground-truth source $P_x$ in a 3D coordinated space and the estimated source position $P_{\hat{x}}$ by:

2 $$\begin{aligned} LocE = ||P_x-P_{\hat{x}}||_2 \end{aligned}$$

To estimate the source position $P_{\hat{x}}$, first, the element-wise power ${{\varvec{\hat{x}}}}^{\cdot 2} = {{\varvec{\hat{x}}}}\cdot {{\varvec{\hat{x}}}}$ is computed from the estimated activity (where A$\cdot$B represents the dot product between A and B). Then, the estimated power is averaged over a defined time-of-interest (TOI). Finally, the position of the source with the highest average power is selected as the estimated location $P_{\hat{x}}$.

This error was used in the proposed methodology as an objective to minimize. Relative error and Pearson Correlation Coefficient were used as additional error measurements to compare the time courses between reconstructions done with different numbers of electrodes. Consider $x_{1i}$ and $x_{2j}$ as the time courses of two reconstructions in the vertices *i* and *j* were the source with highest amplitudes were found, being $x_{1i}$ the time course of the reconstruction computed with the highest number of electrodes. These metrics can be computed by using the following equations:

3 $$\begin{aligned} RelE = \frac{||x_{1i} - x_{2j}||_2}{||x_{1i}||_2} \end{aligned}$$

and

4 $$\begin{aligned} r = \frac{\sum (x_{1i}-\overline{x}_{1i})(x_{2j}-\overline{x}_{2j})}{\sqrt{\sum (x_{1i}-\overline{x}_{1i})^2\sum (x_{2j}-\overline{x}_{2j})^2}} \end{aligned}$$

where $\overline{x}$ represents the mean of the values of *x*.

### Evaluation framework

We evaluated the proposed methodology over synthetic and real EEG signals. In the section below, we present the synthetic EEG dataset with multiple-source activity and its simulation framework that can be used for evaluating inversion algorithms. We have tested the automated methodology for channel selection over this dataset, under single- and multiple-source cases. Furthermore, we have applied the methodology on real EEG signals, by using the dataset “Localize-MI” by Mikulan et al.^25^. HD-EEG was recorded during intracerebral stimulation and the ground-truth activity location is therefore available. Multiple tests of the methodology have been performed and they are presented and described in “Test structure” section.

#### Synthetic EEG dataset

**Figure 2:**
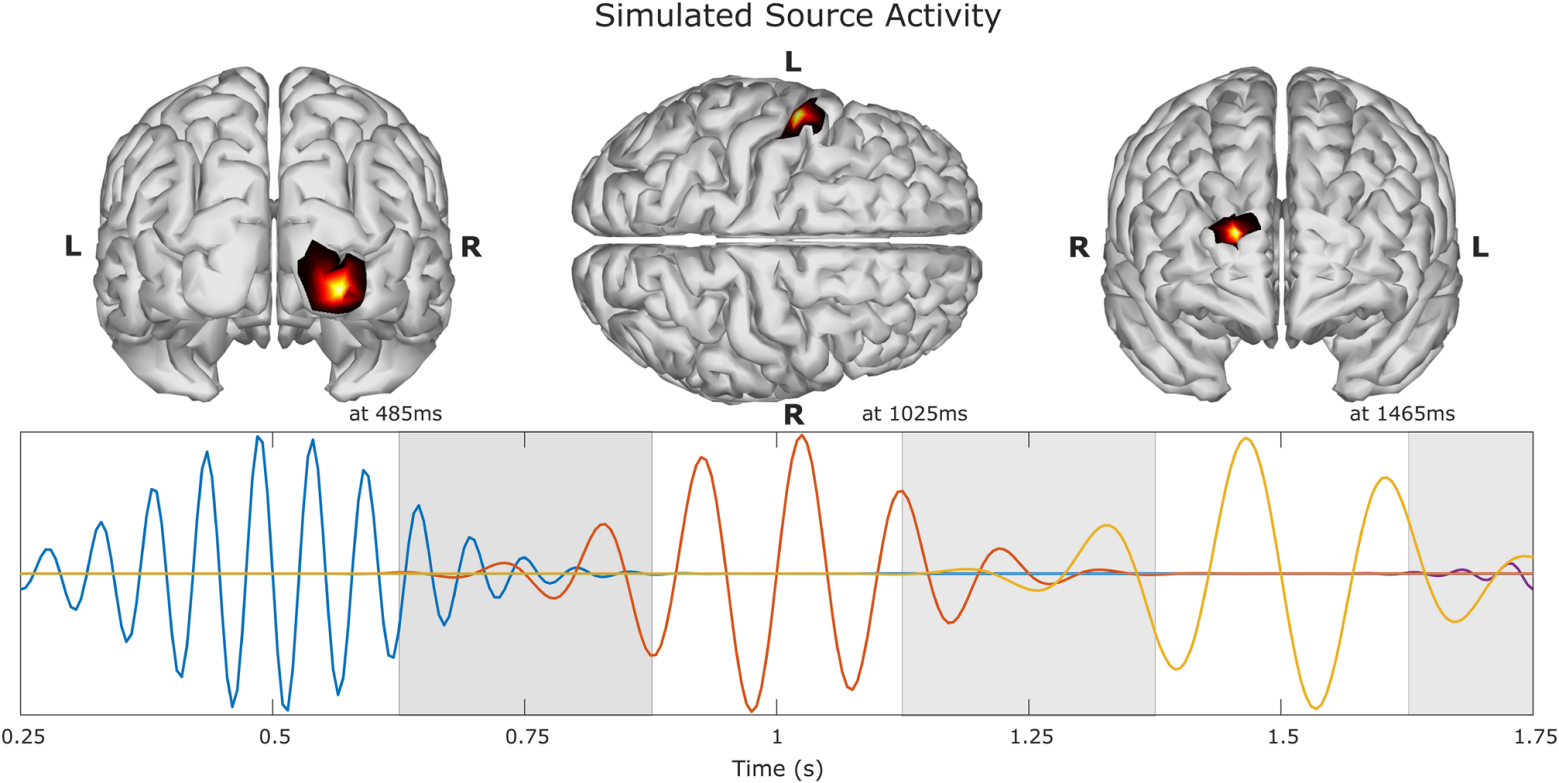
Example of simulated source activity. Source $s_1$ time course is depicted in color blue, source $s_2$ in orange, source $s_3$ in yellow, and the remaining activity from $s_4$ in purple. The locations of the first three sources are depicted on top, where the time was chosen where the maximum amplitude value for each source took place. The light gray areas depict the time sections where there are temporal mixing between sources.

The synthetic EEG dataset consists of 150 trials of multiple source activity. We used the forward equation 1 to simulate the EEG data. Per each trial the sources were distributed over several regions of the brain. The time courses of the sources were generated using a Gaussian windowed sinusoidal^23^, defined by the following equation:

5 $$\begin{aligned} x_i(t_k)=e^{-\frac{1}{2}\left( \frac{t_k-c_i}{\sigma }\right) ^2}sin(2\pi f_it_k) \end{aligned}$$

where *i* represents the source number, $c_i$ the time center of the source activity in seconds, $\sigma _i$ determines the shape of the Gaussian window by adjusting its width, and $f_i$ is the frequency of the sinusoidal activity. The first source $s_1$ was simulated in the occipital lobe. The activity was centered at $c_1=0.5$ s, and the frequency of the activity set at $f_1=19$ Hz in the range of beta rhythm. The second source $s_2$ was located in the sensory-motor cortex, with a time center of $c_2=1$ s, and a frequency of $f_2=10$ Hz in the range of the mu rhythm. The third source $s_3$ was simulated with a frequency of $f_3=7$ Hz in the range of theta rhythm. It was centered in time at $c_3=1.5$ s and located in the frontal lobe. Additional three sources, $s_4$, $s_5$ and $s_6$, were generated with similar parameters (location and frequency) as sources $s_1$, $s_2$, and $s_3$, respectively, but centered at times $c_4=2$ s, $c_4=2.5$ s, and $c_6=3$ s. To maintain the evaluations introduced in “Test structure” section within reasonable limits of computational complexity, only the first three sources of the dataset were considered for testing in this study. An example of the time course of the simulated sources and their location is shown in Fig. 2. For all the sources, the parameter window width was set at $\sigma =0.12$. This value, combined with the center of each source, allowed for temporal mixing between sources, represented by the light gray areas in Fig. 2. Notice that source $s_1$ only has temporal mixing in the transition to the next source, but $s_2$ and $s_3$ have temporal mixing in both sections of the waveform, where around 40% of the source activity is overlapped.

**Figure 3:**
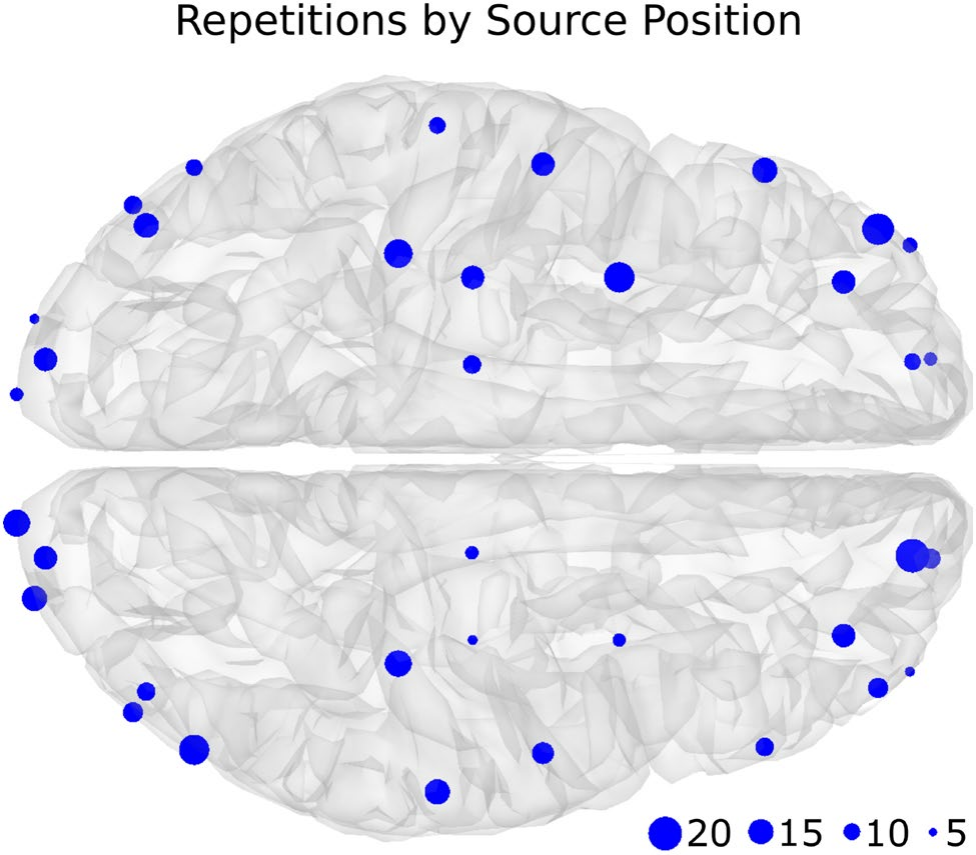
Repetition by source position. Blue circles represent where the simulated activity was located and their diameter represents the number of times that position was selected in the 150 EEG trials. It can be seen that each area (occipital lobe, sensory-motor cortex, and frontal lobe) has six locations per hemisphere.

The sources were distributed over the brain in three main areas, occipital lobe, sensory-motor cortex, and frontal lobe. Per each area, a set of twelve positions (six per hemisphere) was predefined, and during the simulation procedure the position was randomly selected from the pre-defined sets (the information of the 3D location of the sources, the vertex index in the head model, the number of times the source was selected in the dataset and the brain area are presented in the “Supplementary information”  Table 4). This made it possible to create trials with different combinations of sources. Figure 3 shows the locations of the pre-defined sources (represented by the blue circles) and the number of times they were selected in the dataset (represented by the diameter of the circles).

**Figure 4:**
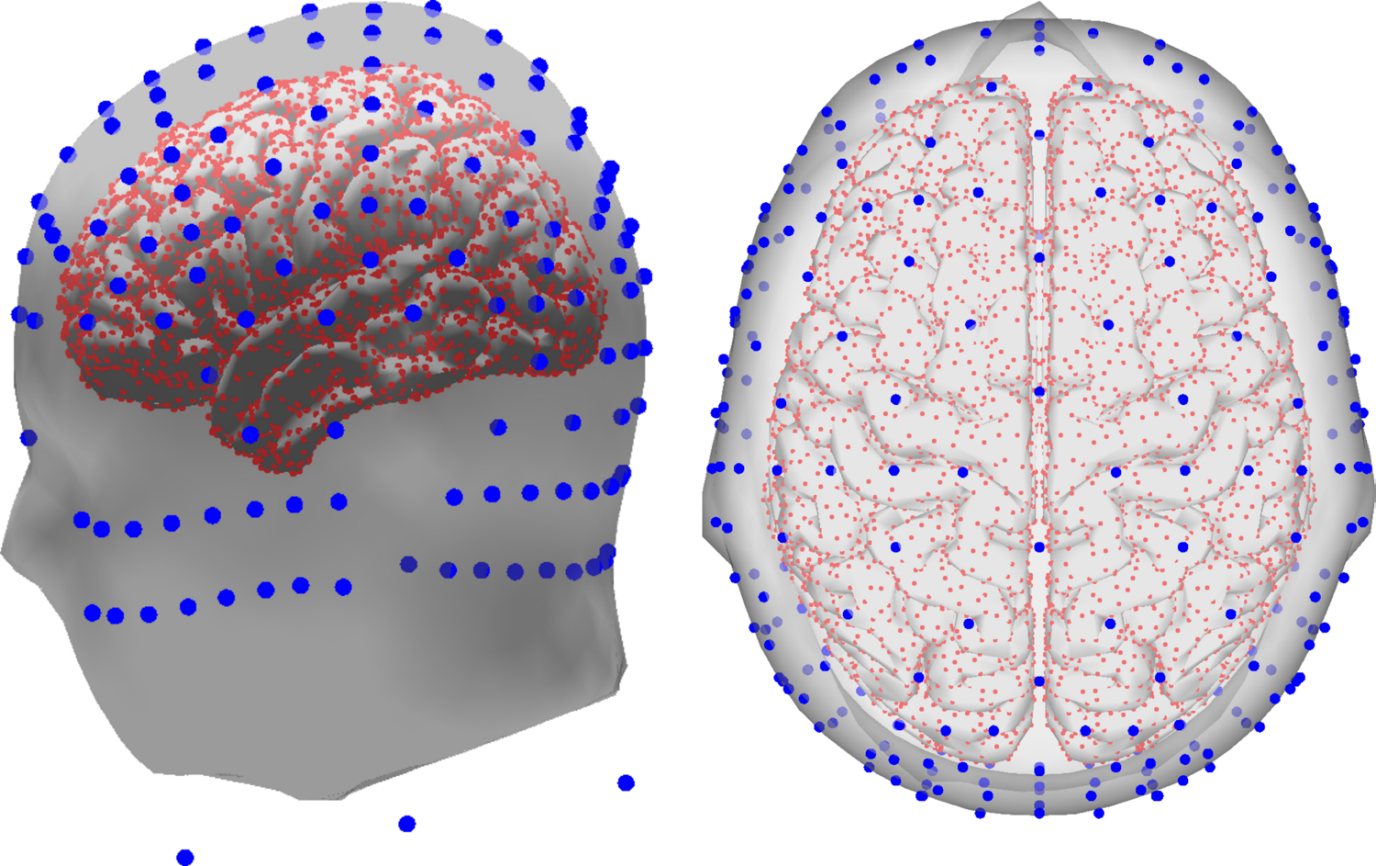
New York head model with 10,000 sources and 231 channels^40^. Lateral view (left) and top view (right). The blue circles represent the location of the EEG channels over the scalp, face, and neck, while the red circles represent the location of the distributed sources on the brain’s cortical areas.

The lead-field matrix for solving the forward problem was taken from a FEM forward model called the “New York head” model^40^. The model is available at https://www.parralab.org/nyhead/. It was computed by modeling the scalp, skull, air cavities, CSF, gray matter, and white matter. In FEM models, the influence of including the CSF and the white matter during modeling has been found significant for solving the inverse problem^41^. The New York head model was proposed as a standard model to be used in EEG studies and it is based on a non-linear average of the MRI of 152 adult human brains. The forward model includes a high-resolution lead field matrix relating 75000 sources with 231 channels. It also includes additional versions with a reduced number of sources of 10000, 5000 and 200 sources. The model considers 231 electrodes, from which 161 are located in the scalp (based on the 10–10 and 10–5 systems), 2 in the left/right preauricular point (LPA/RPA), 4 in the neck, and 64 distributed around the face and in the back of the head below Iz channel. Figure 4 presents a lateral and top view of the channel and source locations using the model with 10000 sources.

The model of 10,000 sources was used for forward computations to generate the EEG signals (see “Synthetic EEG dataset” section) and the model of 5000 sources to calculate the inverse solutions. This allows us to avoid the effects of the so-called inverse crime^42^. The use of the New York head model^40^ in our experiments is based on several reasons: one reason is that the model was computed using FEM including six tissues, and using a more detailed model can yield to more accurate source reconstruction results. Another reason was the number of electrodes available in the model. It considers 231 channels located as per the 10–20 and 10–10 standards and the extended 10–5 system. It is well known that more channels lead to a better reconstruction^11,15^, therefore for a better comparison between the proposed methodology (that identifies an optimized subset of channels) and the full set of electrodes, using the maximum possible number of electrodes is desirable.

After calculating the EEG using Eq. 1, the signals of the channels were corrupted with noise with a signal-to-noise ratio (SNR) of 0 dB (signal and noise with the same power). Each trial had a duration of 3.5 s, with a sampling frequency of 200 Hz. The dataset with the 150 trials and ground-truth information is available at https://github.com/anfesogu/Ground-Truth-EEG-Dataset to facilitate replication of the results in this paper. It can also be used in future studies where a ground-truth for source localization is required.

#### Real EEG dataset

We used the dataset “Localize-MI”^25^ to test the methodology over real EEG signals. The dataset consists of HD-EEG recordings and anonymized MRIs of seven participants that were stimulated with single-pulse biphasic currents by implanted electrodes. The stimulation position is known and the dataset serves as ground-truth for evaluating inverse solutions. The EEG signals were recorded with a 256 geodesic electrode system (Geodesic Sensor Net; HydroCel CleanLeads) where the electrode positions were digitized to allow for co-registration with the MRI. Referring to the original dataset publication: “All of the participants provided their Informed Consent before participating, the study was approved by the local Ethical Committee (protocol number: 463-092018, Niguarda Hospital, Milan, Italy) and it was carried out in accordance with the Declaration of Helsinki.”^25^

The dataset contains a set of pre-processed epochs from − 300 to 50 ms around the stimulation artifact. They were filtered with a high-pass filter at 0.1 Hz, and notch filter at 50, 100, 150, and 200 Hz (only subjects 5 and 7). Baseline correction was applied between − 300 to − 50 ms. Finally the epochs were averaged and cropped 20 ms around the stimulation artifact. In total, the dataset consists of 61 sessions, all of which were further used for testing the methodology. Please refer to the original dataset publication for more detailed information^25^.

A head model of each participant was created to be used during source reconstruction. The models were created by processing the individual MRI using Freesurfer^43^ (Martinos Imaging Centre, Boston, MA) and MNE-python^44^. The number of sources was defined as 4098 per hemisphere, and the lead field matrix was computed using BEM, considering the conductivities of scalp, skull, and brain as 0.3, 0.006 and 0.3 S/m respectively (MNE-python default values).

#### Test structure

In this study we proposed multiple tests to investigate the extent to which the quality of the HD-EEG source reconstruction, measured in terms of source location error, can be retained while using a set of reduced number of channels that were selected by the proposed methodology. The first test was based on source reconstruction for one source. We refer to this as the “single-source test”. This test was performed by setting two minimizing objectives for the optimization: the localization error of the single source and the number of channels. We considered the first source $s_1$ of the synthetic dataset, the epoch was set between 250 to 750 ms, including an overlapping section with the next source $s_2$. In this test, the whole epoch was used as TOI to estimate the source location.

**Figure 5:**
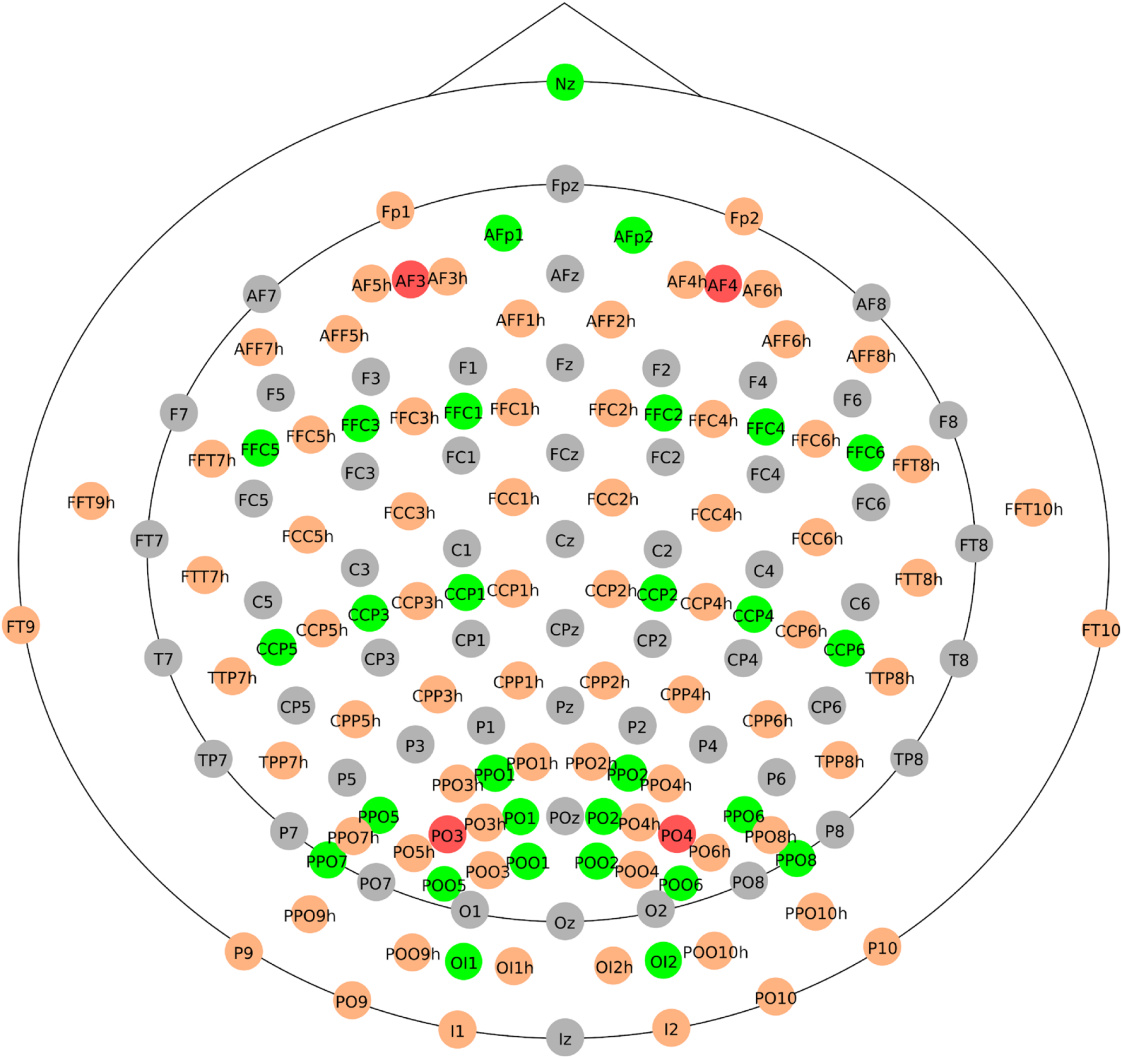
Location and names of the 161 scalp electrodes included in the New York head model^40^. Red and gray circles represent the subset of 60 electrodes, and orange and gray circles represent the subset of 128.

A second test was done considering the first three sources of the synthetic dataset $s_1$, $s_2$, and $s_3$. We refer to this as the “multiple-source test”. In this test, the epoch definition was set between 250 to 1750 ms, and four minimization objectives were set for the optimization algorithm: the individual localization error of each source and the number of channels. The TOIs to estimate the location of the sources were set between 250 to 750 ms, 750 to 1250 ms, and 1250 to 1750 ms for sources $s_1$, $s_2$, and $s_3$, respectively. To evaluate the approach over different numbers of electrodes, we performed three different sub-tests: a first multiple-source sub-test is referred to as “multiple-source test 231e” which considers the full set of 231 electrodes. From those, 161 were located at scalp, and from those 161 electrodes, 73 were located in positions of the standard 10–10 system and 88 were located in positions of the 10–5 system, while the other electrodes were placed in the neck and face areas (Fig. 4). A second multiple-source sub-test referred to as “multiple-source test 128e” constrained the search space of the optimization algorithm to 128 scalp electrodes. From those, 64 electrodes belong to the standard 10–10 and 64 electrodes from the 10–5 system. A third multiple-source sub-test referred to as “multiple-source test 60e” constrained the search space of the NSGA-II to 60 electrodes, all of them located according to the 10–10 standard positions. Figure 5 presents the 161 positions of the scalp electrodes of the New York head model, and the subsets configuration for 128 and 60 electrodes.

The third test was done using the Localize-MI dataset. During each session, the stimulation took place in the same electrode position, therefore, we treated the data as single source case and we set two minimizing objectives for the optimization algorithm: the localization error of the artifact source and the number of channels. We refer to this test as the “Localize-MI test”. In this test, the whole epoch was used as TOI to estimate the source location.

The procedure during all tests started by defining the electrodes to be considered. Only in the multiple-source tests did the electrode number vary according to the test (when constraining the search space to 128 or 60 electrode positions). In the single-source test all the electrodes were considered. In the case of the Localize-MI test, the electrodes that were marked as bad were removed when calculating the forward model and no attempt to clean or interpolate channels was carried out. The number of electrodes used defines the length of the chromosome for the optimization. After setting the number of electrodes, the number of objectives was defined, and the EEG of each trial (in the case of the synthetic data) or the ERP of the session (in the case of the Localize-MI dataset) was transferred to the algorithm. In the next step, the population size and the maximum number of generations were set. Their values were determined experimentally and set as 100 and 400 respectively. Finally, the algorithm for source reconstruction was defined, and the algorithm could start processing data by combining the NSGA-II with sLORETA, wMNE and MSP in separated runs respectively.

Each trial or ERP was processed by the algorithm at least three times, each time with a different source reconstruction algorithm. During a run, the algorithm evaluated 40,000 combinations of electrodes while trying to minimize the objectives. When the run ended, an output file with the combinations and performance indexes of all generations and chromosomes was generated. From this, the performance of the best combination per each number of channels was extracted to create the pseudo-Pareto front. Finally an accuracy index was computed for comparing the reconstructions from the optimized set of channels versus all the channels available. The accuracy index represents the percentage of trials or ERPs that obtained equal or lower localization error with a given number of electrodes when compared to the accuracy when using all the electrodes for the same trial or ERP.

The proposed methodology was implemented and executed in Matlab (The MathWorks, Inc.) version 2016b (www.mathworks.com). The source reconstruction algorithms wMNE and sLORETA were implemented as custom functions in Matlab. To run the MSP method, we used the Statistical Parametric Mapping (SPM; Wellcome Centre for Human Neuroimaging, London, UK) software for Matlab. The NSGA-II solver by^28^ was adapted to include source reconstruction. The tests were carried out using the IDUN computing cluster^45^ of the Norwegian University of Science and Technology NTNU. Parts of Figs. 1, 2, 3, 4, 8, 9, and 10 were generated using Matlab 2016b.

## Results

### Single-source test

**Figure 6:**
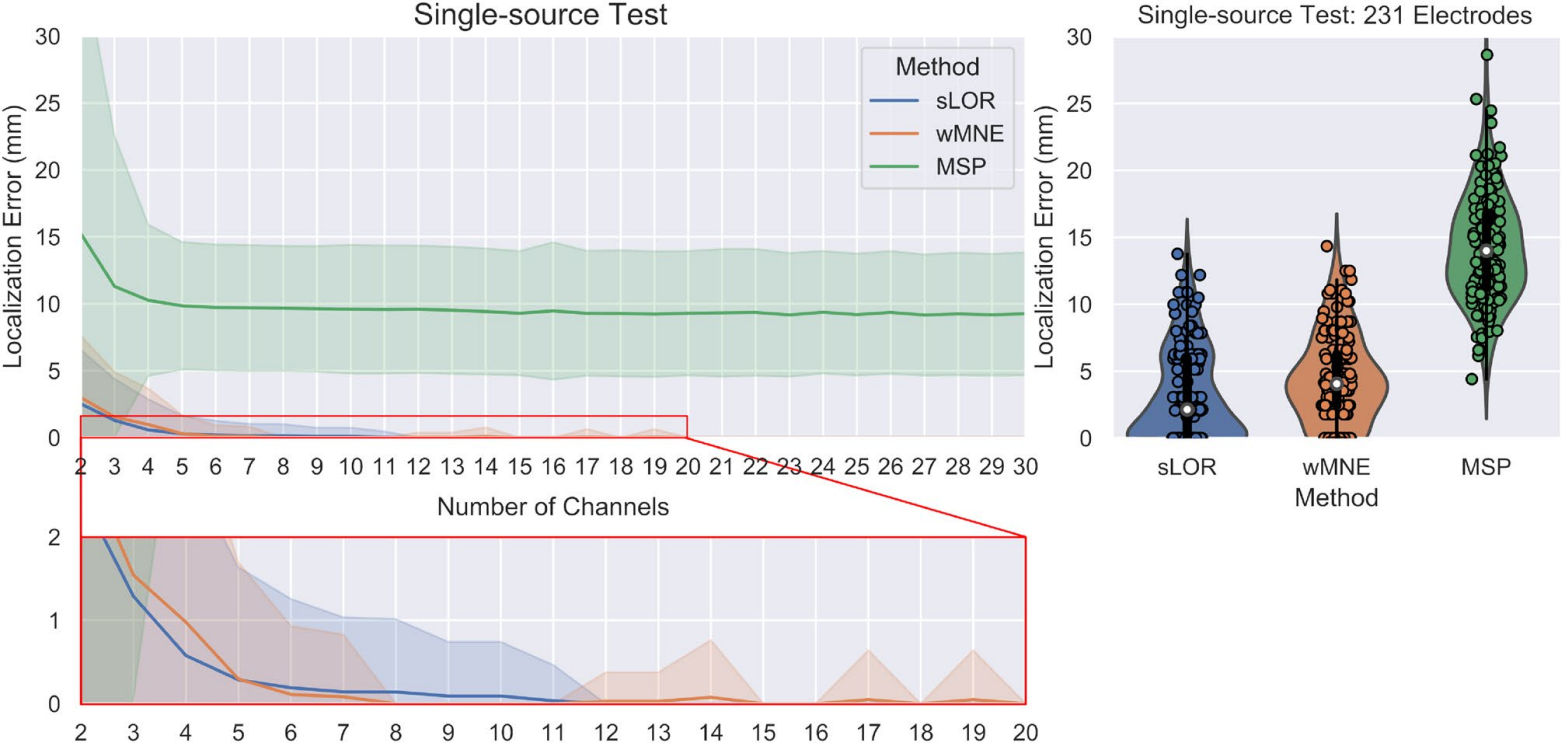
Results for the single-source test. The pseudo-Pareto fronts relating to the number of channels and the localization error by each source reconstruction method are presented at left. The lines represent the mean localization error across the 150 trials and their respective colored bands represent their standard deviation. The mean localization errors across the trials and the standard deviation obtained by using all the electrodes available (231) by each method are presented at right.

The pseudo-Pareto front from the single-source test and localization error with all the electrodes are presented in Fig. 6. The pseudo-Pareto front exhibits a typical convex behavior. The accuracy obtained with the optimized combinations of channels presents a stable value for the localization error when using combinations with five or more channels, independently of the source reconstruction method applied. An inflection point in the pseudo-Pareto front can be seen when reducing from five to four channels. It is also evident when looking at the values presented in Table 1, where the results from the optimization with two to sixteen channels and the results with all the electrodes are summarized. The accuracy index of the optimized combinations of channels was extracted by quantifying the percentage of trials with a given number of channels that obtained equal or lower localization error than when using all the electrodes for the same trial. The accuracy index for the single source test exhibits relatively stable behavior from 16 to 5 channels, but it has a decrease of more than two perceptual points when reducing from five to four channels. Its value continues decreasing substantially while reducing the channels, coinciding with the increasing of the localization error and the standard deviation.

When analyzing the results presented in Fig. 6 and Table 1 on a more detailed level, it can be seen that with a combination of four or more channels, more than 92% of accuracy index was obtained for sLORETA and wMNE, indicating that at least 138 over 150 trials obtained equal or lower accuracy than when using all the electrodes. In the case of MSP it presents a lower accuracy index than the other methods, with a stable value around 88%, and when considering combinations with four or more channels at least 127 of 150 trials were equal or better than the reconstructions with all electrodes.

**Table 1:** Summary of the localization error obtained with the optimized combinations of channels, from 2 to 16, and with the full set of 231 electrodes. The accuracy index represents the percentage of trials with the given number of channels that obtained equal or lower localization error than when using all the electrodes for the same trial.

Method	Chs	Mean loc. error	SD	Acc. index	Method	Chs	Mean loc. error	SD	Acc. Index	Method	Chs	Mean loc. error	SD	Acc. index
sLOR	2	2.5	4.0	66.7	wMNE	2	3.0	4.6	76.7	MSP	2	15.3	21.3	71.3
	3	1.3	3.1	82.7		3	1.5	3.3	89.3		3	11.3	11.2	80.0
	4	0.6	2.3	92.0		4	1.0	2.7	93.3		4	10.3	5.6	84.7
	5	0.3	1.3	95.3		5	0.3	1.4	96.0		5	9.8	4.8	88.0
	6	0.2	1.1	96.7		6	0.1	0.8	98.0		6	9.7	4.7	88.0
	7	0.1	0.9	97.3		7	0.1	0.7	98.7		7	9.7	4.7	87.3
	8	0.1	0.9	97.3		8	0.0	0.0	100.0		8	9.7	4.7	88.7
	9	0.1	0.6	98.0		9	0.0	0.0	100.0		9	9.6	4.7	88.0
	10	0.1	0.6	98.0		10	0.0	0.0	100.0		10	9.6	4.8	88.0
	11	0.0	0.4	99.3		11	0.0	0.0	100.0		11	9.6	4.8	88.7
	12	0.0	0.0	100.0		12	0.0	0.3	99.3		12	9.6	4.7	88.7
	13	0.0	0.0	100.0		13	0.0	0.3	99.3		13	9.5	4.7	88.7
	14	0.0	0.0	100.0		14	0.1	0.7	99.3		14	9.4	4.7	89.3
	15	0.0	0.0	100.0		15	0.0	0.0	100.0		15	9.3	4.6	89.3
	16	0.0	0.0	100.0		16	0.0	0.0	100.0		16	9.5	5.1	88.7
	All	0.0	0.0	–		All	1.6	3.8	-		All	11.8	6.2	–

### Multiple-source test

**Figure 7:**
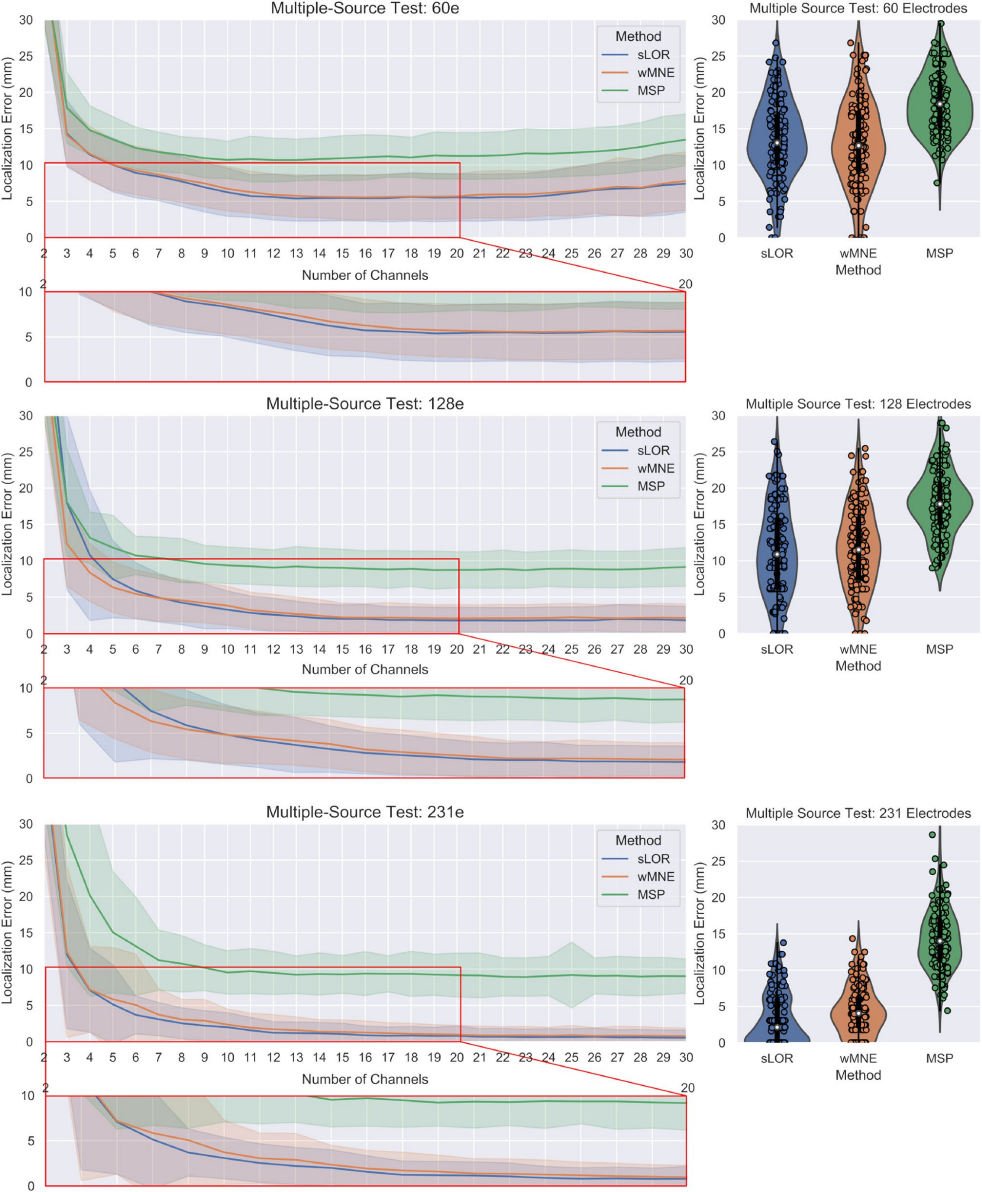
The pseudo-Pareto fronts for the multiple-source test with the different constrained number of channels are shown at left. They relate to the number of channels vs the average between the three sources’ localization error. The lines represent the mean of the localization error$^*$ of the three sources across the 150 trials, and the colored bands the standard deviation. The mean localization error$^*$ across the trials and the standard deviation obtained by using the subsets of 60 and 128 electrodes, and the full set of 231 electrodes for the three sources, are presented at right. $^*$The mean localization error is computed using the average error between the three sources and then the average of this value across the 150 trials.

The pseudo-Pareto fronts for the multiple-source test with the different electrode configurations are shown in Fig. 7. The multiple test had four optimization objectives. To facilitate the visualization of the results, the pseudo-Pareto front was obtained from the mean localization error across the 150 trials, where the localization error is the average of the individual localization error for each one of the three sources. Table 2 presents the localization error, standard deviation and accuracy index for the multiple-source tests for a selected number of channels and with all electrodes.

The results for all electrodes in the three tests show a tendency of increasing the localization error when using a lower number of electrodes (Fig. 7 right column). When localizing the sources, the mean between 60 and 128 channels presents a similar value in each method, with the solution with 60 channels being less accurate. In contrast, the difference is notorious when increasing from 128 to 231 channels, and it is more evident for the sLORETA and wMNE methods. In the pseudo-Pareto’s fronts of the proposed methodology, the same tendency as with all electrodes can be observed. In them, the localization error was more accurate when using the full set of 231 channels, and when restricting the search space to 128 and 60 channels the localization error increased. However, when comparing the solutions for the test with 128 and 231 channels, there is not a considerable difference between the solutions, but the obtained localization error values were lower for sLORETA and wMNE in the multiple-source test with 231 electrodes.

When analyzing the values of table 2, it can be seen that for the multiple-source tests of 60e and 128e, the proposed methodology obtained a lower mean localization error when considering optimized combinations with four or more electrodes than with the 60e and 128e subsets. In the case of the multiple-source test 231e, the proposed methodology obtained lower errors than with all the 231 channels when using optimized combinations of eight or more channels. Regarding the accuracy index, in the multiple-source test 231e, for the optimized combinations with 16 electrodes in 145 of 150 trials, the proposed methodology obtained equal or lower accuracy than the full set of electrodes. The accuracy index reduces as the number of channels decreases, and at the point of eight electrodes the accuracy index presents a value of 58% (87 of 150 trials). However, despite the relatively low accuracy index, the mean across the trials was lower than with the full set.

**Table 2:** Multiple-source test results.

Method	Chs	Mean loc. error	SD	Index (%)	Method	Chs	Mean loc. error	SD	Index (%)	Method	Chs	Mean loc. error	SD	Index (%)
sLOR Test 60e	4	11.40	3.38	65.33	wMNE Test 60e	4	11.53	3.75	58.00	MSP Test 60e	4	14.79	3.36	74.00
	6	8.91	3.43	84.67		6	9.25	3.30	74.67		6	12.34	2.85	90.67
	8	7.73	3.42	91.33		8	8.01	3.15	84.67		8	11.40	2.49	97.33
	10	6.25	3.31	98.00		10	6.72	3.05	92.00		10	10.70	2.56	98.67
	12	5.59	3.16	98.67		12	5.90	2.85	96.00		12	10.68	3.02	98.00
	14	5.45	3.15	98.67		14	5.64	2.91	96.67		14	10.83	2.88	98.00
	16	5.44	3.12	99.33		16	5.55	3.08	98.67		16	11.07	3.10	97.33
	All	13.56	5.25	–		All	13.06	5.77	–		All	18.98	5.07	–
sLOR Test 128e	4	10.75	8.92	55.33	wMNE Test 128e	4	8.35	3.96	69.33	MSP Test 128e	4	13.16	3.54	74.67
	6	5.87	3.84	79.33		6	5.43	3.30	87.33		6	10.72	2.64	94.00
	8	4.23	3.00	87.33		8	4.54	2.90	90.00		8	10.00	3.03	96.67
	10	3.26	2.56	94.00		10	3.82	2.67	96.00		10	9.37	2.51	97.33
	12	2.58	2.25	95.33		12	2.92	2.43	97.33		12	9.04	2.43	96.67
	14	2.13	1.94	98.00		14	2.46	2.18	99.33		14	9.06	2.66	98.00
	16	2.03	1.96	99.33		16	2.20	1.87	100		16	8.90	2.47	98.00
	All	11.28	5.93	–		All	11.74	5.43	-		All	17.82	4.25	–
sLOR Test 231e	4	7.08	5.75	20.00	wMNE Test 231e	4	7.18	5.87	24.00	MSP Test 231e	4	20.15	13.81	29.33
	6	3.69	2.59	44.00		6	5.06	6.99	47.33		6	13.15	6.76	56.00
	8	2.54	2.13	58.00		8	3.06	2.78	60.67		8	10.74	3.87	74.00
	10	2.00	2.00	71.33		10	2.35	2.14	66.67		10	9.53	2.99	82.00
	12	1.23	1.53	88.00		12	1.72	2.00	76.00		12	9.47	2.97	81.33
	14	1.16	1.49	90.00		14	1.38	1.64	82.00		14	9.32	2.90	84.00
	16	0.90	1.26	96.67		16	1.26	1.60	82.67		16	9.39	3.02	86.00
	All	3.17	3.52	–		All	4.43	3.28	-		All	14.19	4.01	–

**Figure 8:**
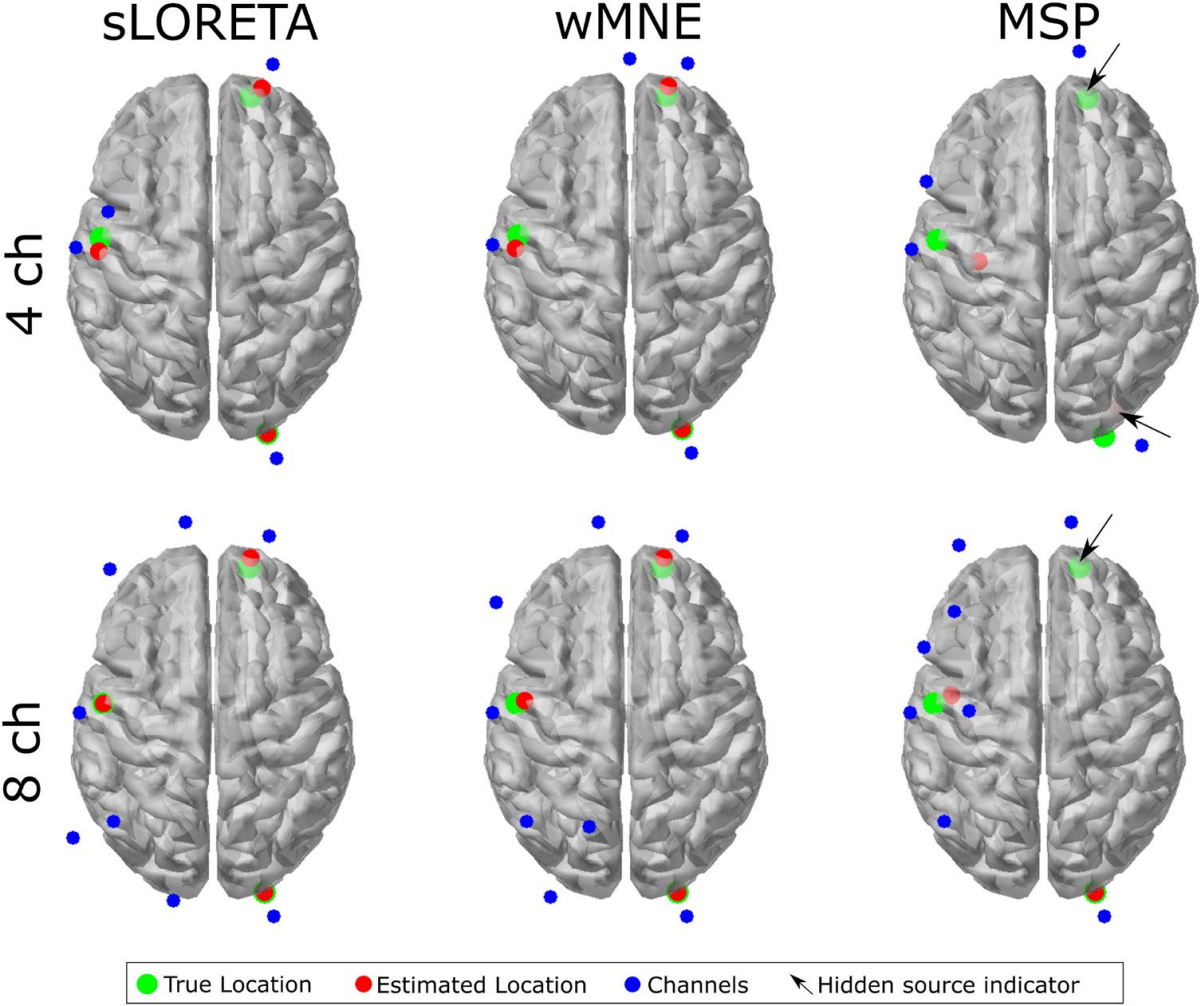
Optimized combinations and source localization for one trial during the multiple-source test 231e. The mean localization errors with four channels were 5.3, 4.7, and 14.5 mm, and with eight channels were 2.4, 4.2, and 4.5 mm, for sLORETA, wMNE, and MSP, respectively. The hidden source indicator is intended to point to where there is a source that can not be seen from the top view.

An example of the position of the true and estimated sources locations and the selected channels is shown in Fig. 8. It shows the optimized combinations with four and eight electrodes for one trial of the dataset, and the resulted combinations were obtained from the multiple-source test 231e results for each source reconstruction method. In the combinations of four channels, it can be observed that the channels selected are located close to the true source location, where the mean localization errors were 5.3 mm, 4.6 mm and 14.5 mm for sLORETA, wMNE, and MSP, respectively. In addition, when observing the combination of eight channels, the selected electrodes were located not only close to the source location, but also in intermediate positions between the sources that were relatively close. In contrast, for the sources that were more separated, there was not selected any channel between them. The mean localization errors for the eight channel combinations were 2.4 mm, 4.2 mm and 4.5mm for sLORETA, wMNE, and MSP, respectively. This suggests that the proposed methodology selected not only the channels that contained the most information from a single source, but also the channels that contained shared information from multiple sources.

**Figure 9:**
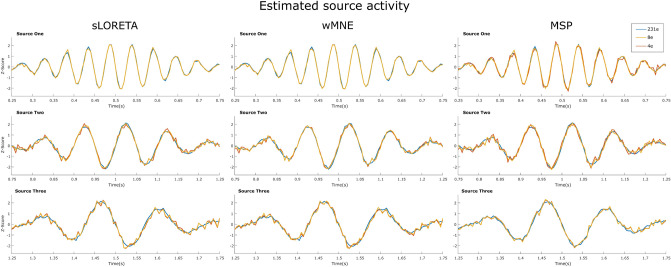
Example of the estimated time courses of the three sources with the full set of 231 channels, and with the optimized combinations with four and eight channels for one trial.

**Figure 10:**
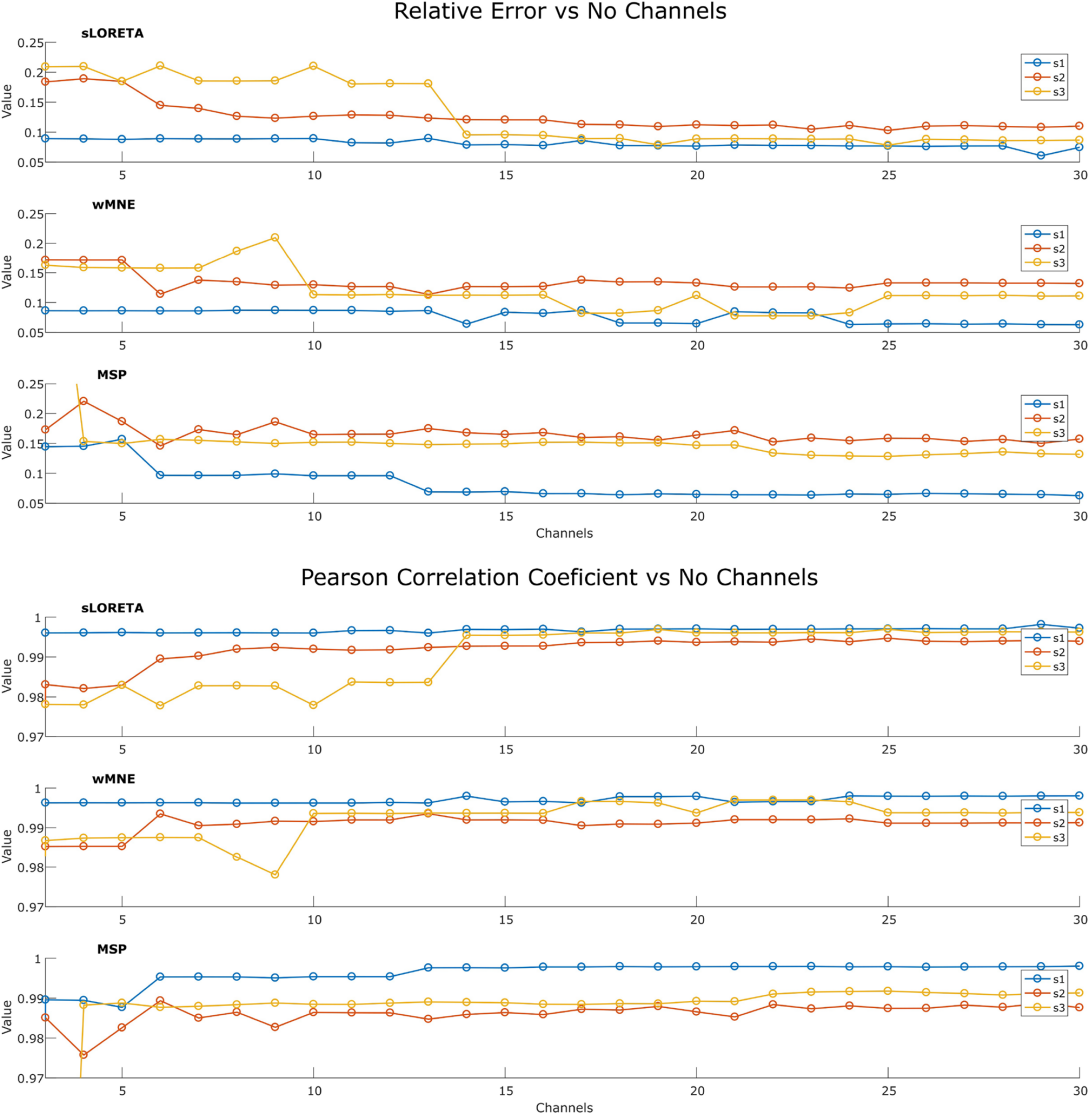
Relative error and Pearson correlation coefficient for each source, comparing the pseudo-Pareto combinations with the full set of 231 channels.

Regarding the effects of the estimated waveform for each source, the standardized time courses of the sources estimated with 231 channels, and with the optimized sets of four and eight channels for the one trial, are presented in Fig. 9. The source waveforms are highly similar independent of the number of channels, however, the reconstructions were noisier when using the optimized sets than with the full set of electrodes. For the same trial, the time courses of the pseudo-Pareto channel combinations were compared against the time course using the 231 channels. The relative error and Pearson correlation coefficient were calculated for each source, each number of channels from 3 to 30, and for each reconstruction algorithm. Its values are presented in Fig. 10. It was found that the source time courses estimated with the optimized combinations and the full set had more than a 97% of correlation when considering five or more channels, obtaining a relative error lower than 0.25. When considering optimized combinations with more electrodes, the difference became smaller. The level of correlation with 16 channels or more is around 99%, independent of the source reconstruction method.

### Localize-MI test

**Figure 11:**
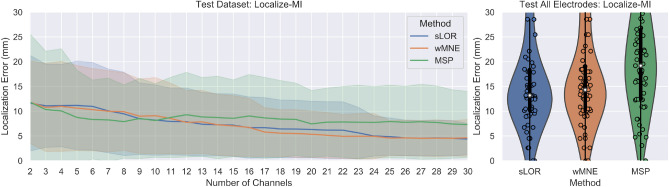
Results for the Localize-MI test. The pseudo-Pareto fronts relating to the number of channels and the localization error by each source reconstruction method are presented at left. The lines represent the mean localization error across the 61 sessions and their respective colored bands represent their standard deviation. The mean localization errors across the trials and the standard deviation obtained by using all the electrodes available (all channels labeled as good) by each method are presented at right.

The ERPs for the 61 sessions were processed by the proposed methodology with each one of the source reconstruction algorithms. During processing, the channels marked as bad channels were not taken into account, neither in the optimization nor in the calculation of the values with “all channels”. The number of channels varied according to the number of channels labeled as good. The average number of good channels among the 61 sessions was 210 channels (sd = 23). In the worst case 162/256 and in the best case 246/256 channels were considered during the optimization process. Therefore, the number of channels used varied between sessions, and the term “all channels” refers here to all channels labeled as good channels. The pseudo-Pareto fronts resulting from processing each one of the ERPs and the values with all channels are presented in Fig. 11. A similar tendency than in the previous tests can be observed, in which the optimized combinations obtained a lower localization error than with all the electrodes. In contrast to previous tests, there is not an exponential trend when considering the fewest number of channels. Instead, a more linear behavior can be observed, where the localization error and standard deviation increases as the number of channels decreases.

Table 3 presents the summary of the localization error, standard deviation and accuracy index for the Localize-MI test for the pseudo-Pareto combinations from two to sixteen channels. The accuracy index shows that more than 70% of the ERPs were located with the same or higher accuracy than with all the electrodes when using 8 or more electrodes, and more than 60% when using four. It is noticeable that with the optimized subset of two channels, the mean localization error was lower than with all channels. In addition, the accuracy index values were 63.93% (39/61) , 65.57% (40/61) , and 40.98% (25/61) for sLORETA, wMNE and MSP respectively, with a marginal difference in the standard deviation for sLORETA and wMNE when compared to the deviation obtained with all electrodes.

**Table 3:** Localize-MI test results.

Method	Chs	Mean loc. error	SD	Index (%)	Method	Chs	Mean loc. error	SD	Index (%)	Method	Chs	Mean loc. error	SD	Index (%)
sLOR	2	11.62	9.57	63.93	wMNE	2	11.82	8.27	65.57	MSP	2	11.83	13.58	40.98
	3	11.10	8.34	63.93		3	10.77	8.83	65.57		3	10.34	11.64	50.82
	4	11.14	8.25	63.93		4	10.99	9.01	65.57		4	10.06	12.41	60.66
	5	11.17	8.90	63.93		5	10.63	8.53	67.21		5	8.74	9.45	70.49
	6	10.98	8.78	63.93		6	10.31	8.24	68.85		6	8.32	7.90	72.13
	7	10.07	8.40	68.85		7	10.00	8.13	68.85		7	8.26	8.35	78.69
	8	9.47	8.35	73.77		8	9.91	7.79	70.49		8	7.91	7.35	81.97
	9	8.49	7.18	73.77		9	9.00	7.44	73.77		9	8.54	8.04	80.33
	10	8.21	7.05	73.77		10	9.11	7.63	73.77		10	8.13	7.39	83.61
	11	7.94	6.70	73.77		11	8.53	7.06	75.41		11	8.75	8.04	78.69
	12	7.89	6.74	73.77		12	7.77	6.35	77.05		12	9.29	8.50	83.61
	13	7.37	6.39	73.77		13	7.84	6.31	75.41		13	8.85	7.89	83.61
	14	7.27	6.23	75.41		14	7.25	6.08	78.69		14	8.72	8.27	88.52
	15	7.18	6.24	75.41		15	7.05	6.06	78.69		15	8.57	7.70	85.25
	16	6.69	6.16	77.05		16	6.74	5.83	80.33		16	9.04	8.30	85.25
	All	15.12	8.88	–		All	15.89	8.94	–		All	19.83	9.61	–

## Discussion

The minimum number of electrodes required for modeling a single source is defined by the number of parameters to describe it: the localization based on three location coordinates (x,y,z) and the orientation based on three strength components on each coordinate axis^46^. However, in distributed models based on FEM and BEM modeling, the orientation can be assumed as fixed throughout the activation period of the source, where the orientation is often assumed to be normal to the cortical surface. Therefore the number of parameters and electrodes required can be reduced. In the single-source test, the results shown in Fig. 6 suggest that four or five channels (where the inflexion point of the pseudo-Pareto front is located) are enough to estimate the source parameters with a satisfying localization accuracy when compared with a set of HD-EEG. These results are also supported by the values shown in Table 3, where the mean localization error was lower with these number of channels than when using all available electrodes.

Previously, it was discussed that a plateau behavior was observed in the localization error when increasing the number of electrodes^15^. This plateau can also be observed in the pseudo-Pareto front for all the tests performed here (Figs. 6, 7 and 11), where adding channels to the subset did not continue decreasing the localization error. However, the plateau did not remain flat until reaching the full number of electrode. At some point adding channels started to increase the localization error. This effect is possible to see in the pseudo-Pareto front of the multiple-source test constrained to 60 channels, where at around 23 channels the error starts increasing notably. This effect is presented because the optimization algorithm was set to identify electrode combinations with the lowest number of electrodes and the lowest localization error possible for each source; during the optimization process, the algorithm does not find the optimal for each number of electrodes, rather, it evaluates a given number and as it finds combinations with a lower number, it keeps the search in that direction. In the end, most of the combinations evaluated lay in the first third of the total electrode number. This effect was also present in the other test but for a higher number of electrodes (not shown in the figures).

The results of the multiple-source tests with 231, 128, and 60 channels evidence the general effect of using a lower number of channels, where the localization error achieved lower values when using 231 channels in both, without optimization when using all channels, and with optimization for the subset of channels. Considering this, when constraining the search to use less electrode positions, as in the tests with 128 and 60 channels, it was possible to observe an evident increment in the localization error with and without optimization (Fig. 7). This accuracy behavior supports the proposition that intermediate positions between electrodes in the 10–10 system adopted by the 10–5 electrode layout play an important role. As there are more channels, the probability of recording closer to the source of interest increases, resulting in a more accurate estimation. Moreover, the results are in line with the widely accepted concept of a higher number of channels leading to a higher localization accuracy, which has been previously demonstrated and discussed^11,15^.

What is new in the results is that an optimized subset of electrodes can attain similar or better localization accuracy than when using all electrodes in a HD-EEG setting (extensions of the 10–20), as shown in Tables 1, 2 for a simulated dataset, and in 3 for the Localize-MI dataset. At first, it can appear counter-intuitive that a reduced number of electrodes can attain such levels of accuracy. However, it should be cautiously noted that not any subset of channels can attain that accuracy. Through the combination of methods proposed in our methodology, it was possible to find combinations of channels for a particular brain source/s configuration that attained values of localization error equal or better than with HD-EEG.

Simplifying the number of electrodes by optimal selection can be beneficial for applications and systems based on a sparse number of channels in medical and non-medical fields. A significant number of EEG devices use less than 32 electrodes, especially in clinical settings, where systems with 21 electrodes (based on the standard 10–20) are still the gold standard for multiple analyses^7^. For those systems, the electrodes can be selected to estimate the activity of an area of interest; as demonstrated, the sources retrieved when using an optimized subset presented a high level of correlation with the signals obtained with the full set of electrodes (Fig. 9). Therefore, the source time-courses can be used for further analysis and feature extraction.

Multiple BCI systems are based on the brain activity and responses of a particular region or area of the brain e.g. classification of steady state visually evoked potential (SSVEP), or motor imaginary movements. Several works have studied the applicability and feasibility of source reconstruction in BCI classification^47,48^ and applied over motor imaginary tasks^49–51^, reporting an increase in the classification accuracy for source-based approaches over the traditional sensor-based methods. We consider that the use of the source space has been poorly exploited in BCI systems, partially due to the uncertainty of source reconstruction when recording with non HD-EEG systems. We believe that a selected configuration of electrodes, based on channel optimization to reconstruct the sources of specific areas, can favor the development of source-centered BCI systems with a sparse number of electrodes that can surpass actual systems in accuracy, portability and comfort.

The use of a low number of electrodes inherently reduces the preparation time and increases the portability of EEG systems. The implementation of the proposed methodology can increase the flexibility of brain source reconstruction in multiple studies. For example, in mobile brain/body imaging (MoBI), it is necessary to make it flexible and ease the acquisition of brain data^52^ in order to perform studies in natural environments for the participants, such as when exercising or working^53^. The proposed methodology also has application in clinical settings in which it is required to monitor the activity of a specific area of the brain. The electrodes required to map a particular region of the brain related to a disease can be identified with our method and placed in a patient, then the estimated time-course can be analyzed, for example, for detecting the start of a seizure in epileptic patients or monitoring changes in brain activation over time.

This optimization-based approach can enable systematic searches of electrode subsets for any given source or combination of sources that can estimate the location/s of the source/s. In addition, the accuracy of different high-density electrode configurations widely used today can be compared for any given source scenario. In low-density systems, it can be used to evaluate the source localization quality of multiple consumer-grade EEG systems that proliferate nowadays^54^; to verify to what extent they can be used, and to determine the potential brain source activity they can monitor.

The use of individual head models can be seen as a particular limitation to extend the applicability of the proposed methodology. We are aware that in multiple applications, especially in non-medical fields, there is no anatomical information available that allows for an estimation of a subject-specific model. However, in such cases it is recommended to use template precise models and to apply a warping process using head landmarks and electrode positions^55^. After warping the model and co-registering with the electrodes, the forward model can be computed and used during source reconstruction. Another limitation of the study is the use of sources at the cortical level. Further studies should be performed to evaluate to what extent the methodology can be used for reconstructing deep source activity, as it has been proven to be detectable by HD-EEG source imaging^56^.

EEG connectivity analysis is a growing topic nowadays, as it offers the possibility to measure and quantify the interaction of brain regions during multiple tasks^57^. The study of brain connectivity is proven to be useful for neuromarker identification of different brain conditions and processes^58–60^. The source reconstructed activity offers localized and unmixed information that can be used to perform connectivity analysis between brain regions^61,62^. However, this analysis on the source space has been limited to the use of HD-EEG. In^63^ the impact of using HD-EEG (with 256, 128, and 64 electrodes) and LD-EEG (with 32 electrodes) for reconstructing and detecting resting state networks (RSNs) was evaluated. It was found that the influence of the electrode count is higher in deeper brain networks than the default mode network (DMN). In contrast, minimal differences between LD-EEG and HD-EEG were found in detecting other networks such as the dorsal somatomotor network (DSN) or medial prefrontal network (MPN). In the future, it would be interesting to evaluate if the proposed methodology can identify optimal electrode combinations in LD-EEG montages that can detect such networks with HD-EEG accuracy.

In conclusion, the different tests performed in this study offer multiple views of the channel optimization effects for source reconstruction in which multiple- and single-source cases were analyzed. Overall, the proposed methodology was able to optimize the localization error and the number of channels, by finding a subset of channels with few electrodes that attained equal or similar localization accuracy than HD-EEG. In both cases, when analyzing the Localize-MI and the synthetic EEG datasets, the sources have different localization, which allowed us to evaluate the methodology over different brain regions and source configurations. Additionally, the proposed methodology was tested on several electrode systems (10–20, 10–10, 10–5, and geodesic) and considering different head modeling methods (FEM and BEM). Independently of the variations, our proposed methodology was able to find combinations of channels with few electrodes that offer equal or better localization accuracy, demonstrating the feasibility of using low-density optimized electrode combinations to localize single and multiple sources.

## Supplementary Information

Supplementary Information.

## Data Availability

The synthetic dataset of multiple-source activity analyzed in this document can be downloaded from https://github.com/anfesogu/Ground-Truth-EEG-Dataset, where an example code for generating a synthetic dataset is also available. The forward model used for generating the synthetic EEG dataset and solving the inverse problem “New York head”^40^ model is available at https://www.parralab.org/nyhead/. The dataset of Simultaneous human intracerebral stimulation and HD-EEG “Localize-MI”^25^ can be found in https://doi.gin.g-node.org/10.12751/g-node.1cc1ae/. The NSGA-II solver^28^ can be found in https://se.mathworks.com/matlabcentral/fileexchange/31166-ngpm-a-nsga-ii-program-in-matlab-v1-4.
