# Supplementary material for: Automated methodology for optimal selection of minimum electrode subsets for accurate EEG source estimation based on Genetic Algorithm optimization
Source: Sci Rep. 2022 Jul 2;12:11221. doi: 10.1038/s41598-022-15252-0 (PMC9250504; doi:10.1038/s41598-022-15252-0)
Supplement: Supplementary file 1 — Supplementary Information. [file 41598_2022_15252_MOESM1_ESM.pdf]

4 Supplementary Material

4.1 Source location in the simulated dataset

Table 4. 3D location, vertex index, repetition and brain area information of the simulated sources related to the New York head model use for the simulated dataset.

X	Y	Z	Vertex Index	Repetition	Brain Area
-62.43	-27.28	49.89	266	10	Sensory-motor
-54.55	-72.80	24.21	700	10	Occipital
-54.03	34.01	13.48	712	15	Frontal
-55.18	-7.500	51.86	764	14	Sensory-motor
-43.65	-81.75	30.91	1180	15	Occipital
-47.53	-84.24	6.520	1205	11	Occipital
-39.92	61.19	-11.14	1600	9	Frontal
-42.97	55.24	14.43	1784	19	Frontal
-33.91	6.770	65.33	1973	18	Sensory-motor
-33.05	48.80	34.39	2153	14	Frontal
-38.35	-34.58	70.96	2271	17	Sensory-motor
-33.89	-20.66	73.05	2284	14	Sensory-motor
-26.13	-102.69	-8.060	2734	6	Occipital
-18.64	65.05	-12.77	3228	8	Frontal
-17.57	-20.79	78.50	3422	11	Sensory-motor
-18.52	-100.63	20.50	3461	14	Occipital
-18.12	61.65	14.96	3511	10	Frontal
-12.01	-106.02	6.470	3727	8	Occipital
62.43	-27.28	49.89	5274	15	Sensory-motor
54.55	-72.80	24.21	5708	18	Occipital
54.03	34.01	13.48	5720	11	Frontal
55.18	-7.500	51.86	5772	13	Sensory-motor
43.65	-81.75	30.91	6188	11	Occipital
47.53	-84.24	6.520	6213	12	Occipital
39.92	61.19	-11.14	6608	6	Frontal
42.97	55.24	14.43	6792	12	Frontal
33.91	6.770	65.33	6981	8	Sensory-motor
33.05	48.80	34.39	7161	14	Frontal
38.35	-34.58	70.96	7279	16	Sensory-motor
33.89	-20.66	73.05	7292	6	Sensory-motor
26.13	-102.69	-8.060	7742	15	Occipital
18.64	65.05	-12.77	8236	12	Frontal
17.57	-20.79	78.50	8430	8	Sensory-motor
18.52	-100.63	20.50	8469	14	Occipital
18.12	61.65	14.96	8519	20	Frontal
12.01	-106.02	6.470	8735	16	Occipital
